# L-AmB as the Real First-Line Therapy for Paediatric Visceral Leishmaniasis: A Multicentre Retrospective Study from an Endemic Area of Southern Italy

**DOI:** 10.3390/antibiotics15090904

**Published:** 2026-09-14

**Authors:** Ottavia Avola, Daniela Cuzzubbo, Martina Cinnirella, Lucia Gozzo, Piera Samperi, Maria Licciardello, Vito Miraglia, Filippo Greco, Martino Ruggieri, Silvia Marino, Milena La Spina, Mattia Comella, Antonino Trizzino, Andrea Di Cataldo, Giovanna Russo

**Affiliations:** 1 Department of Clinical and Experimental Medicine, University of Catania, 95123 Catania, Italy; ottaviaavola@gmail.com (O.A.); cinnirellamartina18@gmail.com (M.C.); m.ruggieri@unict.it (M.R.); adicata@unict.it (A.D.C.); 2 Paediatric Onco-Haematology Unit, Azienda Ospedaliero Universitaria Policlinico “Rodolico-San Marco”, Via Santa Sofia 78, 95123 Catania, Italy; danielacuzzubbo@hotmail.com (D.C.); psamperi@unict.it (P.S.); marinella72@icloud.com (M.L.); vitomiraglia@hotmail.com (V.M.); 3 Health Department, AOU “Policlinico G. Rodolico-San Marco”, Via Santa Sofia 78, 95123 Catania, Italy; l.gozzo@policlinico.unict.it; 4 Unit of Clinical Paediatrics, Department of Medical and Paediatric Sciences, University of Catania, Via Santa Sofia 78, 95123 Catania, Italy; filippo.greco@policlinico.unict.it; 5 Unit of Paediatrics and Paediatric Emergency, AOU “Policlinico G. Rodolico-San Marco”, Viale Carlo Azeglio Ciampi, 95121 Catania, Italy; silvia_marino86@hotmail.it (S.M.); mlaspina@unict.it (M.L.S.); 6 Department of Paediatric Haematology and Oncology, ARNAS Ospedale Civico Di Cristina Benfratelli Hospital, Piazza Nicola Leotta 4, 90127 Palermo, Italy; mattiacomella@gmail.com (M.C.); trizzino@hotmail.com (A.T.)

**Keywords:** visceral leishmaniasis, *Leishmania infantum*, liposomal amphotericin B, pentavalent antimonials, paediatrics, real-world evidence, Mediterranean region

## Abstract

**Background/Objectives:** visceral leishmaniasis (VL) remains an important zoonotic disease in endemic Mediterranean regions, where children represent one of the populations at highest risk of developing symptomatic infection. Although pentavalent antimonials have historically been considered first-line therapy for VL, their use in Italy has become increasingly limited because of restricted availability, safety concerns, and the widespread adoption of liposomal amphotericin B (L-AmB). **Methods:** this multicentre retrospective observational study evaluated the contemporary management of paediatric VL in three tertiary referral centres in Catania, Southern Italy, between January 2016 and December 2025. **Results:** 19 children were included, with a mean age of 4.13 years. The most common clinical manifestations were prolonged fever, pancytopenia, and hepatosplenomegaly. Three different L-AmB regimens were identified, with the 10 mg/kg/day for two consecutive days schedule representing the most frequently adopted protocol. L-AmB was well tolerated; three patients (15.8%) experienced disease relapse during follow-up, all of whom responded successfully to additional L-AmB treatment, resulting in an overall clinical cure rate of 100%. Notably, no patient received pentavalent antimonials, reflecting their complete replacement in routine clinical practice. **Conclusions:** these findings provide real-world evidence that L-AmB has effectively become the contemporary first-line treatment and standard of care for paediatric VL in an endemic Mediterranean setting, highlighting the discrepancy between historical therapeutic paradigms and current clinical practice.

## 1. Introduction

Visceral leishmaniasis (VL), also known as kala-azar, is the most severe clinical manifestation of *Leishmania* infection and remains one of the world’s most important neglected tropical diseases [1]. Despite a substantial decline in global incidence over the last decade, VL continues to cause considerable morbidity and mortality, with an estimated 50,000–90,000 new cases occurring annually [2,3]. In fact, VL cases are declining globally, but cutaneous leishmaniasis (CL) is increasing in the Eastern Mediterranean Region [4]. As of July 2026, 62 VL-endemic countries (78%) and 61 CL-endemic countries (67%) had reported data to the WHO Global Leishmaniasis Programme for 2024. In 2024, approximately 85% of global VL cases were reported from seven countries: Brazil, Ethiopia, India, Kenya, Somalia, South Sudan, and Sudan. Seven countries—Afghanistan, Algeria, Brazil, Colombia, Iran (Islamic Republic of), Peru, and the Syrian Arab Republic—each reported more than 5000 CL cases and collectively accounted for 83% of the global reported CL incidence. Globally, 152 imported cases of CL and 519 imported cases of VL were reported in 2024 [5]. In 2023, WHO recognised Bangladesh as the first country to be validated for VL elimination as a public health issue. VL is endemic in over 70 countries across tropical, subtropical and Mediterranean regions. Its epidemiology is influenced by the complex interaction between parasite species [5]. In the Mediterranean basin, *Leishmania infantum* is the predominant etiological agent, and transmission occurs through a zoonotic cycle in which domestic dogs represent the main reservoir [6,7]. Italy remains one of the major endemic countries in Southern Europe, with Sicily representing one of the areas with the highest and most persistent circulation of *L. infantum* [8].

Interestingly, the specific epithet *infantum*, derived from the Latin *infans* (“unable to speakchild”), reflects the historical description of the parasite in paediatric patients, highlighting the close association between this pathogen and childhood disease that still characterizes Mediterranean VL today. Indeed, children are particularly susceptible to developing symptomatic VL, especially during the first years of life, when cell-mediated immunity is still maturing. The disease usually presents with prolonged fever, hepatosplenomegaly, pancytopenia, and hypergammaglobulinemia, although the initial clinical picture is frequently nonspecific [9]. Consequently, paediatric VL often enters the differential diagnosis of haematological malignancy, autoimmune disease, hemophagocytic lymphohistiocytosis, and other severe infectious conditions [10]. Because untreated VL is almost invariably fatal, maintaining a high index of clinical suspicion in children living in or returning from endemic areas remains essential [10]. Diagnosis is traditionally based on the demonstration of *Leishmania* amastigotes in tissue aspirates. Although splenic aspiration provides the highest diagnostic sensitivity, approaching 95–99%, its invasive nature and the potential risk of haemorrhagic complications, particularly in patients with marked splenomegaly and thrombocytopenia, limit its routine use in clinical practice. Consequently, bone marrow aspiration remains the reference diagnostic procedure in most paediatric centres, while molecular techniques, particularly real-time polymerase chain reaction (RT-PCR), have progressively improved diagnostic sensitivity and now play an increasingly important role in routine clinical practice [11].

The therapeutic approach to VL has changed profoundly over the past two decades. Pentavalent antimonials, once considered the standard treatment throughout the Mediterranean region, have progressively disappeared from routine paediatric practice because of their unfavourable safety profile, prolonged treatment schedules, parenteral administration, contraindications in several clinical settings, and limited availability in many European countries [12]. Conversely, L-AmB has consistently demonstrated excellent efficacy, rapid parasite clearance, favourable tolerability, and remarkably low relapse rate. These characteristics have established L-AmB as the preferred therapeutic option for paediatric VL in high-income countries, where it is currently recommended as the standard of care by international guidelines.

Although the superiority of L-AmB has been well established, contemporary evidence describing its implementation in routine clinical practice remains surprisingly limited. Most published studies have evaluated therapeutic efficacy, compared different dosing regimens, or reported single-centre experiences, whereas multicentre real-world studies reflecting the current management of paediatric VL in endemic Mediterranean regions remain scarce. Furthermore, many reviews continue to present pentavalent antimonials as first-line therapeutic options, reflecting traditional treatment paradigm rather than contemporary paediatric practice. This discrepancy between guideline recommendations, the published literature, and daily clinical management highlights the need for updated real-world evidence describing how paediatric VL is currently diagnosed and treated in endemic European settings.

Eastern Sicily offers a unique epidemiological scenario for addressing this knowledge gap. As one of the Italian regions where *L. infantum* remains endemic, it provides the opportunity to evaluate the contemporary management of paediatric VL within referral centres that routinely diagnose and treat this disease. Therefore, the present multicentre retrospective study aimed to describe the epidemiological, clinical, laboratory, microbiological, therapeutic, and outcome characteristics of children diagnosed with VL between 2016 and 2025 in three tertiary paediatric hospitals in Catania. In particular, we sought to document the current therapeutic landscape of paediatric VL, evaluating the real-world use of L-AmB as the contemporary first-line treatment and describing the progressive disappearance of pentavalent antimonials from routine clinical practice in an endemic Mediterranean area.

## 2. Results

### 2.1. Study Population

A total of 19 paediatric patients diagnosed with VL between January 2016 and December 2025 were included in the study.

**Figure 1:**
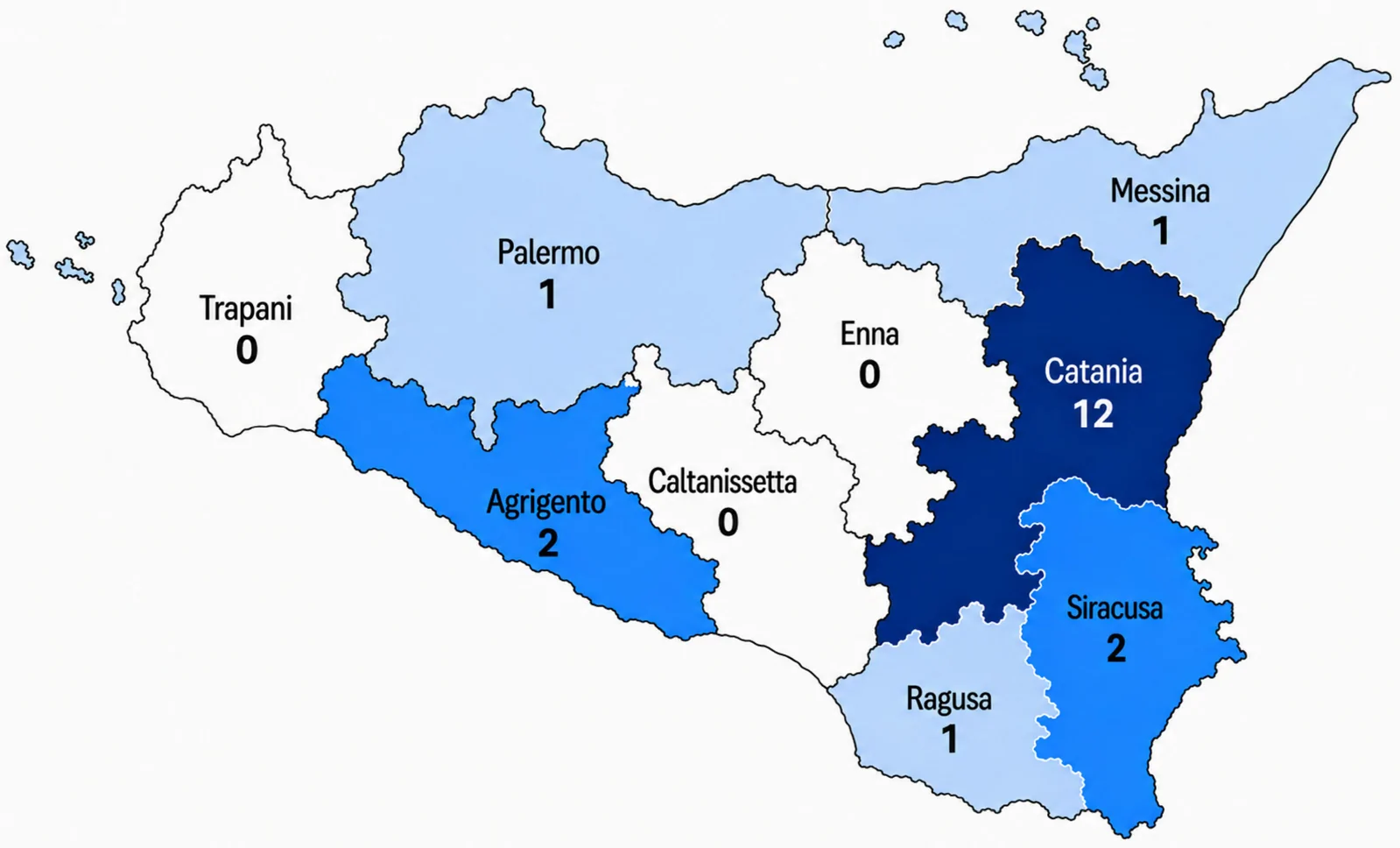
Provincial distribution of the study population. Choropleth map showing the geographic origin of the 19 paediatric patients diagnosed with VL between 2016 and 2025 in Eastern Sicily. Colour intensity reflects the number of cases in each province, with darker shades indicating a higher number of cases.

The main demographic characteristics of the study population are reported in Table 1 and Figure 1.

### 2.2. Clinical Presentation

The most frequent presenting symptoms and physical findings are summarized in Table 2.

### 2.3. Laboratory and Microbiological Findings

Baseline laboratory abnormalities are summarized in Table 3. Anaemia was the only laboratory finding observed in all patients, whereas leukopenia and thrombocytopenia were less frequent. Elevated inflammatory markers and hypergammaglobulinemia were common, while hypertransaminasemia was documented in approximately 40% of the cohort.

### 2.4. Diagnostic Methods

Diagnosis was established through an integrated diagnostic approach combining serological, parasitological, and molecular investigations (Table 4). Anti-*Leishmania* antibodies were detected in all patients (19/19), providing initial evidence supporting the clinical suspicion of VL. Bone marrow aspiration was performed in all cases and represented the cornerstone of the diagnostic work-up. Microscopic examination identified *Leishmania* amastigotes in 16 of 19 patients (84.2%), whereas three patients (15.8%) had negative bone marrow microscopy despite a high clinical suspicion. In these cases, the diagnosis was confirmed by RT-PCR performed on bone marrow samples, which was positive in all three patients. Overall, RT-PCR was performed in seven patients (36.8%), yielding positive results in all tested cases.

The mean interval between hospital admission and definitive diagnosis was 8.1 ± 8.5 days (median 8.0 days, range 0–36). The longest diagnostic delay occurred in a child initially managed for macrophage activation syndrome (MAS). Notably, the prolonged diagnostic interval in this patient was largely attributable to the initial hyperinflammatory presentation, which closely mimicked MAS and was subsequently recognized as an HLH-like syndrome triggered by VL. Nevertheless, the shortest interval (0 days) was observed in an immunodeficient patient undergoing immediate bone marrow examination because of suspected haematological malignancy. In our cohort, however, we could not demonstrate that diagnostic delay adversely affected treatment response or final clinical outcome, since all patients ultimately achieved clinical cure.

### 2.5. Treatment

Treatment characteristics are summarized in Table 5.

Baseline characteristics according to treatment regimen are summarized in Supplementary Table S1. Descriptive analysis showed substantial clinical heterogeneity within treatment groups, with no clear pattern suggesting that longer treatment schedules were preferentially selected for patients with greater baseline disease severity. Notably, patients receiving the 10 mg/kg/day, 2-day regimen included children with marked thrombocytopenia and extensive splenomegaly. Because of the small and markedly unbalanced treatment groups, these findings were considered descriptive, and no inferential comparisons were performed.

L-AmB demonstrated an excellent safety profile. A transient rise in serum creatinine was seen in a single immunodeficient patient receiving extended therapy, but no clinically significant treatment-related adverse events occurred.

### 2.6. Clinical Outcome

Clinical outcome is summarized in Table 5.

Overall, L-AmB achieved clinical cure in all patients included in the study, corresponding to an overall treatment success rate of 100%. Sixteen patients achieved complete remission after a single treatment course, whereas three experienced disease relapses. Importantly, all relapsing patients responded successfully to additional courses of L-AmB, with no need for alternative antileishmanial therapy. Furthermore, treatment was well tolerated, with only one transient episode of increased serum creatinine observed in an immunodeficient patient receiving an extended therapeutic regimen.

Among the three patients who experienced relapse, two were immunocompetent children. The first was a 1.5-year-old boy who presented with severe thrombocytopenia (platelet count 5970/mm^3^) and hypergammaglobulinemia. Bone marrow examination demonstrated *Leishmania* amastigotes by microscopy and was also positive by RT-PCR, and the diagnosis was established four days after hospital admission. He received L-AmB at 10 mg/kg/day for two consecutive days and experienced relapse two months after treatment. The second was a 1.73-year-old girl presenting with anaemia and thrombocytopenia, in whom bone marrow microscopy and RT-PCR were both positive. Diagnosis was established two days after hospital admission. She received L-AmB at 3 mg/kg/day for 5 consecutive days plus an additional dose on day 10 and experienced relapse four months after treatment. Both patients were successfully retreated with L-AmB at 3 mg/kg/day for 10 consecutive days, with subsequent clinical resolution.

## 3. Discussion

In this multicentre retrospective study, L-AmB demonstrated excellent effectiveness and a favourable safety profile for paediatric VL (VL) in an endemic area of Southern Italy. The demographic characteristics of our cohort were consistent with the epidemiology of Mediterranean VL, which predominantly affects young children. Although the overall mean age was 4.13 years, exclusion of the single 20-year-old immunodeficient patient yielded a mean age of 3.2 years. Most cases originated from the province of Catania, reflecting both local endemicity and the referral role of our tertiary paediatric centres.

Clinical presentation was relatively homogeneous, with fever and splenomegaly being almost universal and anaemia observed in all patients, while hepatomegaly, inflammatory marker elevation, hypergammaglobulinemia, leukopenia, and thrombocytopenia occurred with variable frequency. However, VL was rarely the initial diagnostic suspicion, as most children were investigated for cytopenias, persistent fever with organomegaly, or unexplained inflammatory manifestations. This clinical overlap with haematological and hyperinflammatory disorders highlights the diagnostic complexity of paediatric VL and the role of tertiary referral centres in providing appropriate diagnostic evaluation. Beyond bone marrow infiltration and hypersplenism, anaemia has also been attributed to inflammation-driven disturbances of iron metabolism and impaired incorporation of iron into the heme molecule, resulting in ineffective erythropoiesis and reduced haemoglobin synthesis [13]. Three cases with atypical presentation were observed. One adolescent underwent emergency splenectomy for traumatic splenic rupture, with retrospective histological examination subsequently demonstrating L. infantum infection, highlighting the importance of considering VL in patients with unexplained splenomegaly in endemic areas. In addition, two patients presented with HLH-like manifestations; in one case, the initial presentation was interpreted as MAS and treated with intravenous immunoglobulins before VL was recognized as the underlying trigger. These cases highlight the remarkable diagnostic overlap between VL and hyperinflammatory syndromes, including HLH, MAS, and Kawasaki-like presentations, which may delay appropriate antiparasitic therapy [14,15].

VL should therefore be systematically considered in the differential diagnosis of children presenting with otherwise unexplained fever, cytopenias, hepatosplenomegaly, and laboratory features suggestive of HLH, particularly in endemic areas [14]. Prompt recognition of the underlying parasitic infection is essential, as specific antiparasitic treatment alone is often sufficient to reverse the inflammatory syndrome and avoid unnecessary immunomodulatory therapies [16].

Despite the endemic setting, diagnosis remained challenging, with a mean interval of eight days from admission. The longest delay occurred in a child initially presenting with MAS, whereas immediate diagnosis was achieved in an immunodeficient patient investigated for suspected acute leukaemia, highlighting how overlapping haematological and hyperinflammatory presentations may influence diagnostic timing. Although anti-*Leishmania* serology was positive in all patients, this should not be interpreted as 100% diagnostic sensitivity, and serology alone was not considered sufficient for definitive diagnosis. Bone marrow microscopy identified *Leishmania* amastigotes in 16/19 patients, while RT-PCR confirmed the three microscopy-negative cases, supporting the complementary role of parasitological and molecular methods, although its availability may itself contribute to diagnostic delay. In our tertiary referral setting, bone marrow aspiration should not be regarded solely as a confirmatory test for VL. Children frequently present with fever, cytopenias, organomegaly, or hyperinflammatory features that overlap with haematological malignancies and HLH/MAS, making bone marrow examination relevant to the differential diagnosis. Moreover, serology provides indirect evidence of infection and may be affected by cross-reactivity, whereas bone marrow examination allows direct parasitological confirmation and provides material for molecular testing when microscopy is inconclusive.

In our setting, samples for RT-PCR are referred to the regional reference laboratory in Palermo, introducing additional logistical constraints. Thus, serological, parasitological, and molecular approaches should be considered complementary rather than alternative, and microbiological confirmation should be pursued whenever feasible. Current international recommendations advocate for a stepwise diagnostic strategy based on clinical suspicion followed by serological and/or molecular investigations, reserving tissue sampling for selected cases. In many endemic areas, rapid diagnostic tests based on the recombinant rK39 antigen have become the preferred first-line investigation because of their simplicity and high diagnostic accuracy [1]. In contrast, the diagnostic pathway adopted in our centres relied primarily on clinical suspicion, anti-*Leishmania* serology, bone marrow aspiration, and targeted RT-PCR, reflecting the diagnostic resources and routine clinical practice of tertiary paediatric referral hospitals in Southern Italy.

Three main L-AmB regimens were used during the study period: 10 mg/kg/day for two consecutive days, 3 mg/kg/day for ten days, and 3 mg/kg/day for five consecutive days plus an additional dose on day 10; two additional patients received individualized schedules, including one immunodeficient patient requiring an extended regimen. Although different dosing schedules were adopted over the study period, the short-course regimen consisting of 10 mg/kg/day for two consecutive days represented the preferred therapeutic approach in more than half of the patients, reflecting the progressive tendency toward simplified treatment schedules whenever clinically appropriate [17,18]. It may be supposed that the preference for a shorter schedule is due to the young age of the patients, making it difficult to maintain safe intravenous access for several days.

The overall relapse rate observed in our cohort was 15.8% (3/19). One relapse occurred in the immunodeficient patient receiving an extended therapeutic regimen, a clinical setting in which impaired host immunity may contribute to an increased risk of recurrence. The other two relapses occurred in immunocompetent children following different L-AmB schedules: one occurred two months after the short-course regimen of 10 mg/kg/day for 2 consecutive days, whereas the other occurred four months after the conventional regimen of 3 mg/kg/day for 5 consecutive days plus an additional dose on day 10. Both patients responded successfully to retreatment with L-AmB at 3 mg/kg/day for 10 consecutive days.

Although the observed relapse proportion may appear relatively high, it should be interpreted cautiously given the small sample size, as each individual event substantially affects the calculated percentage. Importantly, relapses were not confined to the two-day short-course regimen, and our data therefore do not provide evidence of reduced durability of response specifically associated with this schedule. However, the small and unbalanced treatment groups preclude meaningful comparisons of relapse risk across regimens. These findings highlight the importance of adequate post-treatment follow-up irrespective of the L-AmB schedule adopted, while larger multicentre studies are needed to assess the comparative long-term efficacy of different treatment regimens.

L-AmB demonstrated an excellent safety profile. Apart from a transient increase in serum creatinine observed in the immunodeficient patient receiving prolonged therapy, no clinically relevant treatment-related adverse events were documented. This finding is consistent with the extensive experience accumulated in paediatric onco-haematology setting, where L-AmB has long represented one of the safest and most effective antifungal agents in highly vulnerable immunocompromised patients. Such familiarity has likely contributed to its progressive adoption as the preferred treatment for paediatric VL in everyday clinical practice. Our findings are consistent with previous paediatric reports from Italy and other endemic countries, which have consistently shown that L-AmB provides high cure rates, rapid clinical improvement, and excellent tolerability [19]. Nevertheless, the limited sample size does not allow meaningful comparisons between regimens, and larger multicentre studies are needed to establish whether shorter schedules provide comparable long-term efficacy.

Despite its established role in paediatric VL, L-AmB remains an off-label treatment for this indication in Italy, requiring informed parental consent and institutional authorization. Conversely, pentavalent antimonials, although historically regarded as standard therapy, are not routinely available and generally require importation, potentially delaying treatment initiation. This creates a discrepancy between regulatory status and contemporary clinical practice, in which L-AmB has effectively become the preferred therapeutic option. Italian legislation allows off-label prescribing under defined conditions when supported by adequate evidence and when appropriate therapeutic alternatives are unavailable. Given the established efficacy and safety of L-AmB and the limited availability of licensed alternatives, regulatory and reimbursement pathways more closely aligned with current clinical practice could facilitate timely and consistent access to treatment.

The present study has several limitations. Its retrospective design and relatively small sample size limit the possibility of comparing different therapeutic regimens or identifying predictors of relapse. Furthermore, management strategies evolved during the study period according to local clinical practice. Nevertheless, the multicentre design and the inclusion of referral paediatric hospitals from one of the main endemic areas in Southern Italy provide a representative snapshot of contemporary clinical practice. Our findings support the concept that L-AmB has effectively become the standard of care for paediatric VL in this setting, while pentavalent antimonials now play only a marginal role in routine clinical management.

## 4. Materials and Methods

### 4.1. Study Design and Setting

A retrospective observational study was carried out using administrative data gathered from January 2016 to December 2025. The data came from the Departments of Paediatric Haematology and Oncology and Paediatrics at the Azienda Ospedaliero-Universitaria Policlinico “G. Rodolico-San Marco” in Catania, Italy. This included the Paediatric Haematology and Oncology Unit (Policlinico “G. Rodolico”), the Paediatric Clinic (Policlinico “G. Rodolico”), and the Paediatric Unit of San Marco Hospital.

The study focused on the hospital use of L-AmB prescribed for the treatment of paediatric VL as an off-label indication according to Italian Law No. 94/1998. Administrative data routinely collected by the Health Department for institutional monitoring of off-label prescriptions were retrospectively analysed.

Given the small overall sample size and the markedly unbalanced number of patients across treatment regimens, no inferential comparisons or multivariable adjustment between treatment groups were performed, as these analyses would not provide reliable estimates. Baseline demographic, clinical, and laboratory characteristics were therefore summarized descriptively according to L-AmB treatment regimen to explore potential differences in baseline disease characteristics and treatment selection. These comparisons were considered exploratory and were not intended to establish comparative efficacy or causality.

### 4.2. Data Collection

The data collection was carried out for administrative purposes, to control and monitor off-label use in the hospital setting. Administrative records were reviewed to identify all paediatric patients who received L-AmB for VL during the study period.

For each patient, the following variables were collected: age, sex, year of treatment, prescribing centre, therapeutic indication, L-AmB treatment regimen, cumulative dose, duration of therapy, route of administration, length of hospital stay, occurrence of relapse, and other available outcome measures. Additional clinical variables, including presenting symptoms (fever, hepatosplenomegaly, lymphadenopathy), laboratory findings (pancytopenia, hypergammaglobulinemia), microbiological diagnosis (bone marrow microscopy, RT-PCR, or both), and supportive treatments were extracted from the institutional administrative database whenever available.

Patients were eligible for inclusion if VL was diagnosed in the presence of a compatible clinical presentation and positive anti-*Leishmania* serology, together with parasitological and/or molecular confirmation. Specifically, diagnosis was considered confirmed by either microscopic identification of *Leishmania* amastigotes in bone marrow aspirates or detection of *Leishmania* DNA by RT-PCR on bone marrow samples. Accordingly, patients with negative bone marrow microscopy were included only when molecular testing confirmed the diagnosis.

Although anti-*Leishmania* serology was positive in all patients included in our cohort, serological positivity alone was not considered sufficient for definitive diagnosis, as antibody-based assays may have limited specificity because of potential cross-reactivity and do not provide direct evidence of active parasitic infection. Therefore, parasitological and/or molecular confirmation was required for inclusion in the study.

The different therapeutic regimens adopted during the study period included:

L-AmB 10 mg/kg/day for 2 consecutive days;

L-AmB 3 mg/kg/day for 10 consecutive days;

L-AmB 3 mg/kg/day for 5 consecutive days followed by an additional 3 mg/kg dose on day 10.

### 4.3. Ethical Considerations

The present study consisted exclusively of a retrospective analysis of anonymized administrative data that had been routinely collected for institutional monitoring of off-label drug prescriptions within the hospital setting. Data collection and analysis were performed for administrative and pharmacovigilance purposes without any intervention in patient management.

According to the Italian Medicines Agency (AIFA) Guideline for the Classification and Conduct of Observational Studies, ethics committee approval was not required because the study involved secondary analysis of anonymized data collected during routine clinical practice for administrative purposes. No additional procedures were performed for research purposes, and no directly identifiable patient information was processed.

### 4.4. Statistical Analysis

Descriptive statistics were used to summarize patient characteristics, treatment regimens, and clinical outcomes. Categorical variables were expressed as absolute frequencies and percentages, whereas continuous variables were reported as mean ± standard deviation (SD) or median and interquartile range (IQR), according to data distribution.

Statistical analyses were performed using Microsoft Excel 2021 (Microsoft Corporation, Redmond, WA, USA).

## 5. Conclusions

This multicentre real-world study confirms that L-AmB represents an effective, safe, and well-tolerated treatment for paediatric VL in an endemic area of Southern Italy. Rapid clinical improvement, a low relapse rate, and the absence of significant treatment-related toxicity observed in our cohort further support its central role in the management of this potentially life-threatening disease.

Our findings also highlight the discrepancy between current clinical practice and the regulatory framework in Italy. Although L-AmB is widely recognized as the therapeutic standard for paediatric VL and is routinely adopted in referral centres, its use remains off label, while licensed antileishmanial alternatives are not readily available. This situation emphasizes the need for regulatory pathways that better reflect contemporary scientific evidence and facilitate timely access to the most appropriate treatment.

Finally, the sustained endemic circulation of *Leishmania infantum* in Southern Italy underscores the importance of maintaining a high index of clinical suspicion, promoting early diagnosis, and implementing integrated control strategies targeting both human disease and animal reservoirs [20]. Further multicentre studies involving larger paediatric populations are warranted to optimize treatment regimens and strengthen the evidence supporting L-AmB as the standard of care for paediatric VL in endemic Mediterranean settings.

## Figures and Tables

**Table 1:** Demographic and epidemiological characteristics of the study population.

Variable	Value
Total patients n	19
Sex	
Female	13 (68.4%)
Male	6 (31.6%)
Age at diagnosis (years)	
Mean ± SD	4.13 ± 5.97
Range	0.42–20
Referral centre	
Paediatric Haematology and Oncology Unit	12/19 (63.2%)
Paediatric Clinic	4/19 (21%)
Paediatric Unit, San Marco Hospital	3/19 (15.8%)

**Table 2:** Clinical presentation of paediatric patients with VL at hospital admission (n = 19). HLH/MAS: Hemophagocytic Lymphohistiocytosis/MAS.

Clinical Characteristic	Value
Fever	19 (100.0%)
Duration of fever before diagnosis, days, mean ± SD	14.1 ± 12.6
Splenomegaly	18 (94.7%) ^†^
Below the umbilical transverse line	6 (33.3%)
Above the umbilical transverse line	12 (66.6%)
Hepatomegaly	12 (63.2%)
Lymphadenopathy	6 (31.6%)
HLH-/MAS-like presentation	2 (10.5%)

^†^ One patient had previously undergone splenectomy following traumatic splenic injury.

**Table 3:** Baseline laboratory findings of paediatric patients with VL at hospital admission. Continuous variables are reported as mean ± standard deviation (SD), median (interquartile range, IQR), and range, whereas categorical variables are expressed as number (percentage).

Variable	Mean ± SD	Median (IQR)	Range	Abnormal
Haemoglobin	8.01 ± 1.65 g/dL	8.3 g/dL	5.4–10.9 g/dL	19 (100%)
WBC *	5139 ± 2577/mmc^3^	5090/mmc^3^	1370–11,480/mmc^3^	4 (21.1%)
Platelets ^†^	107,223 ± 67,750/mmc^3^	98,000/mmc^3^	4590–267,000/mmc^3^	12 (63.1%)
C reactive protein	66.3 ± 61.9 mg/L	51.1 mg/L	2.57–228 mg/L	18 (94.7%)
IgG ^‡^	2567 ± 2047 mg/dL	1615 mg/L	371–6636 mg/L	12/13 (92.3%)
AST §	/	63 (47–133) U/L	35–415 U/L	8 (42.1%)
ALT §	/	41.5 (21.25–67.75) U/L	9–405 U/L	8 (42.1%)

* Leukopenia defined as WBC < 4000/mm^3^. ^†^ Thrombocytopenia defined as platelet count <150,000/mm^3^. ^‡^ IgG values were available for 13 patients only. § Hypertransaminasemia was defined as elevation of AST and/or ALT above the age-adjusted upper reference limit.

**Table 4:** Diagnostic methods used in the study cohort. Diagnosis was established through an integrated diagnostic approach combining serological, parasitological, and molecular investigations. Bone marrow aspiration was performed in all patients, whereas RT-PCR was mainly used to confirm cases with negative or inconclusive microscopic examination.

Diagnostic Test	Patients Tested (n/N)	Positive (no./no. Tested)
Anti-*Leishmania* serology	19/19	19/19
Bone marrow microscopy	19/19	16/19
RT-PCR on bone marrow aspirate	7/19	7/7

**Table 5:** Treatment regimens and clinical outcomes in paediatric patients with VL treated with L-AmB. Treatment schedules, relapse rates, clinical cure, and treatment-related adverse events are summarized for each therapeutic regimen.

Treatment Regimen	Patients	Relapses	Clinical Cure	Adverse Events
L-AmB 10 mg/kg/day for 2 days	11 (57.9%)	1 (9.1%)	11 (100%)	0
L-AmB 3 mg/kg/day for 10 days	2 (10.5%)	0	2 (100%)	0
L-AmB 3 mg/kg/day for 5 days + day 10	4 (21.1%)	1 (25%)	4 (100%)	0
L-AmB 3 mg/kg/day for 5 days	1 (5.3%)	0	1 (100%)	0
Extended regimen *	1 (5.3%)	1 (100%)	1 (100%)	1 (100%)
Overall	19 (100%)	3 (15.8%)	19 (100%)	1 (5.3%)

* Immunodeficient patient treated with an extended regimen.

## Data Availability

The data supporting the findings of this study are available from the corresponding author upon reasonable request.

## Supplementary Materials

The following supporting information can be downloaded at: https://www.mdpi.com/article/10.3390/antibiotics15090904/s1, Table S1: Baseline characteristics according to L-AmB treatment regimen: data are presented as median (IQR) for groups containing more than one patient and as individual values for treatment groups containing a single patient. Categorical variables are reported as n/N available because of missing baseline data. * One patient in this group had previously undergone splenectomy and was therefore excluded from the assessment of splenomegaly extent.
