# Supplementary material for: L-AmB as the Real First-Line Therapy for Paediatric Visceral Leishmaniasis: A Multicentre Retrospective Study from an Endemic Area of Southern Italy
Source: Antibiotics (Basel). 2026 Sep 14;15(9):904. doi: 10.3390/antibiotics15090904 (PMC13603420; doi:10.3390/antibiotics15090904)
Supplement: Supplementary file 1 [file antibiotics-15-00904-s001.zip › antibiotics-4471617-supplementary.pdf]

Table S1. Baseline characteristics according to L-AmB treatment regimen: data are presented as median (IQR) for groups containing more than one patient and as individual values for treatment groups containing a single patient. Categorical variables are reported as no./no. available because of missing baseline data. *One patient in this group had previously undergone splenectomy and was therefore excluded from the assessment of splenomegaly extent.

**Characteristic**	**10 mg/kg/day for 2 days (no. 11)**	**3 mg/kg/day for 10 days (no. 2)**	**3 mg/kg/day for 5 days + day 10 (no. 4)**	**3 mg/kg/day for 5 days (no. 1)**	**Extended regimen (no. 1)**
Age, years, median (IQR)	0.91 (0.89–1.37)	2.11 (2.08–2.14)	7.03 (1.40–13.50)	0.89	20.0
Haemoglobin, g/dL, median (IQR)	7.75 (6.38–8.93)	9.55 (9.48–9.63)	7.80 (7.65–7.95)	5.4	10.9
Platelet count, /mm^3, median (IQR)	102,000 (65,000–124,000)	128,500 (100,750–156,250)	94,000 (49,295–180,500)	87,000	114,000
Splenomegaly above the umbilical transverse line, n/N available	8/10	2/2	2/3*	0/1	0/1
Hepatomegaly, n/N available	7/10	0/2	3/4	1/1	1/1
